# Supplementary material for: Beiging of perivascular adipose tissue regulates its inflammation and vascular remodeling
Source: Nat Commun. 2022 Sep 7;13:5117. doi: 10.1038/s41467-022-32658-6 (PMC9452496; doi:10.1038/s41467-022-32658-6)
Supplement: Supplementary file 1 — Supplementary Information [file 41467_2022_32658_MOESM1_ESM.pdf]

Supplementary Information

Beiging of perivascular adipose tissue regulates
its inflammation and vascular remodeling

Adachi Y., *et al*

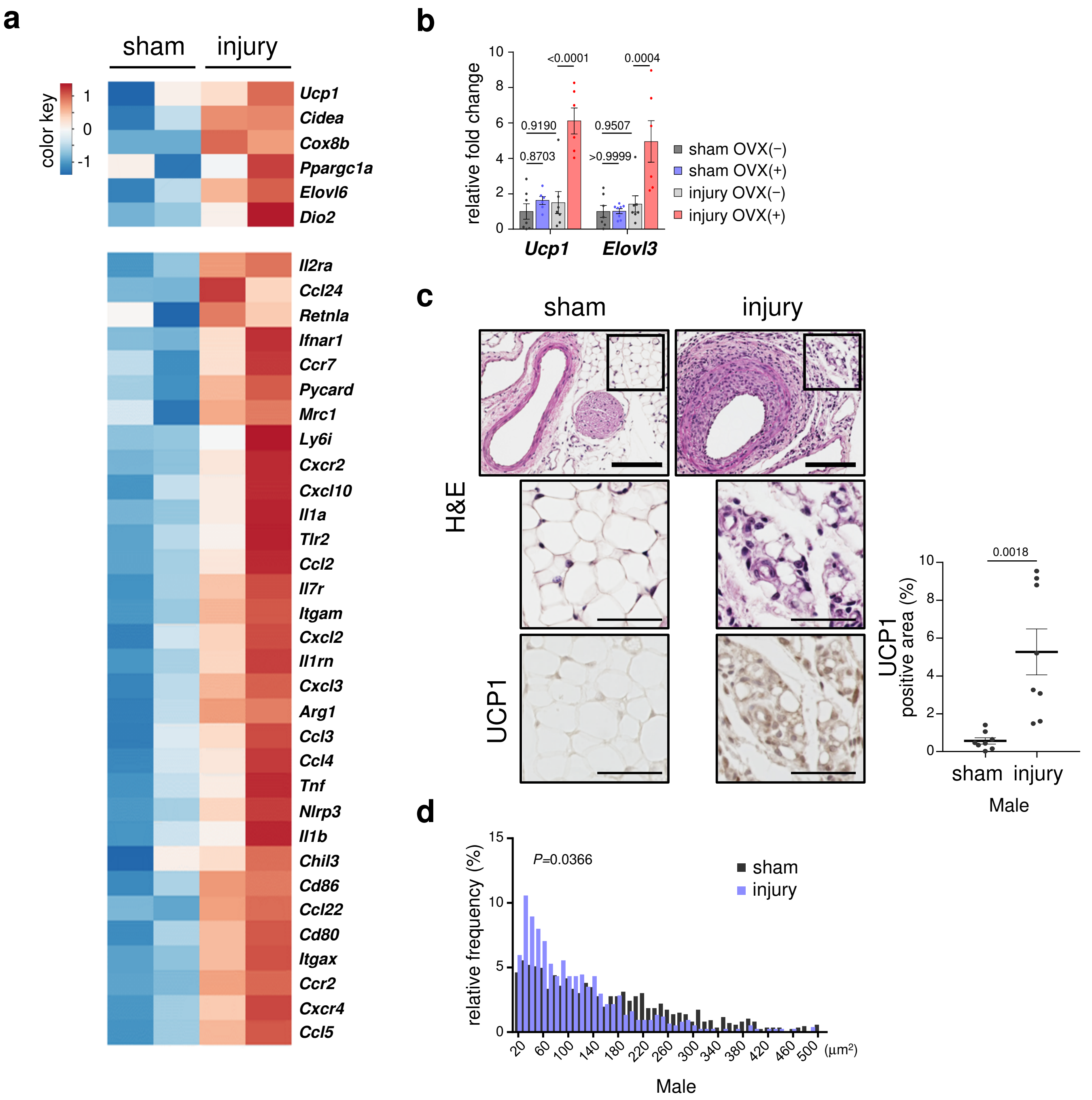

Supplementary Fig. 1 | PVAT inflammation and beiging after endovascular injury.
(**a**) Heat map of results of RNA sequencing analysis showing the upregulated expression of brown adipose tissue (BAT)/beige marker genes (top) and genes encoding immune cell markers and inflammatory cytokines (bottom) in perivascular adipose tissue (PVAT) surrounding femoral arteries (FAs) at 48 h after injury as compared with that of sham-operation. (**b**) Gene expression of BAT/beige markers (*Ucp1* and *Elovl3*) in PVAT 24 h after injury with or without ovariectomy (OVX) (*Ucp1*, n = 7, 6, 7, 6, respectively; *Elovl3*, n = 7, 8, 7, 6, respectively, one-way ANOVA followed by Tukey–Kramer post-hoc test). (**c**) H&E and immunohistochemical staining for UCP1 in injured (14 days after injury) or sham-operated FAs and the outer tissue of wild type male mice. Representative images are shown. Scale bars represent 100 µm (thick bars) and 50 µm (thin bars). The positive area of immunostaining in PVAT was analyzed (n = 8 for each group, unpaired two-tailed Student's *t*-test). (**d**) Histograms of adipocyte area of sham-operated or injured PVAT (14 days after injury) of wild type male mice. Three images of each biological replicate were analyzed and combined to create the histogram. The size distribution between each group was compared using Kolmogorov-Smirnov test. Each bin was normalized to a percent of the total count for that individual tissue. Adipocytes of the bin size in the range of 20-500 µm² were included for the analysis. Data represent mean ± SEM. Source data are provided as a Source Data file.

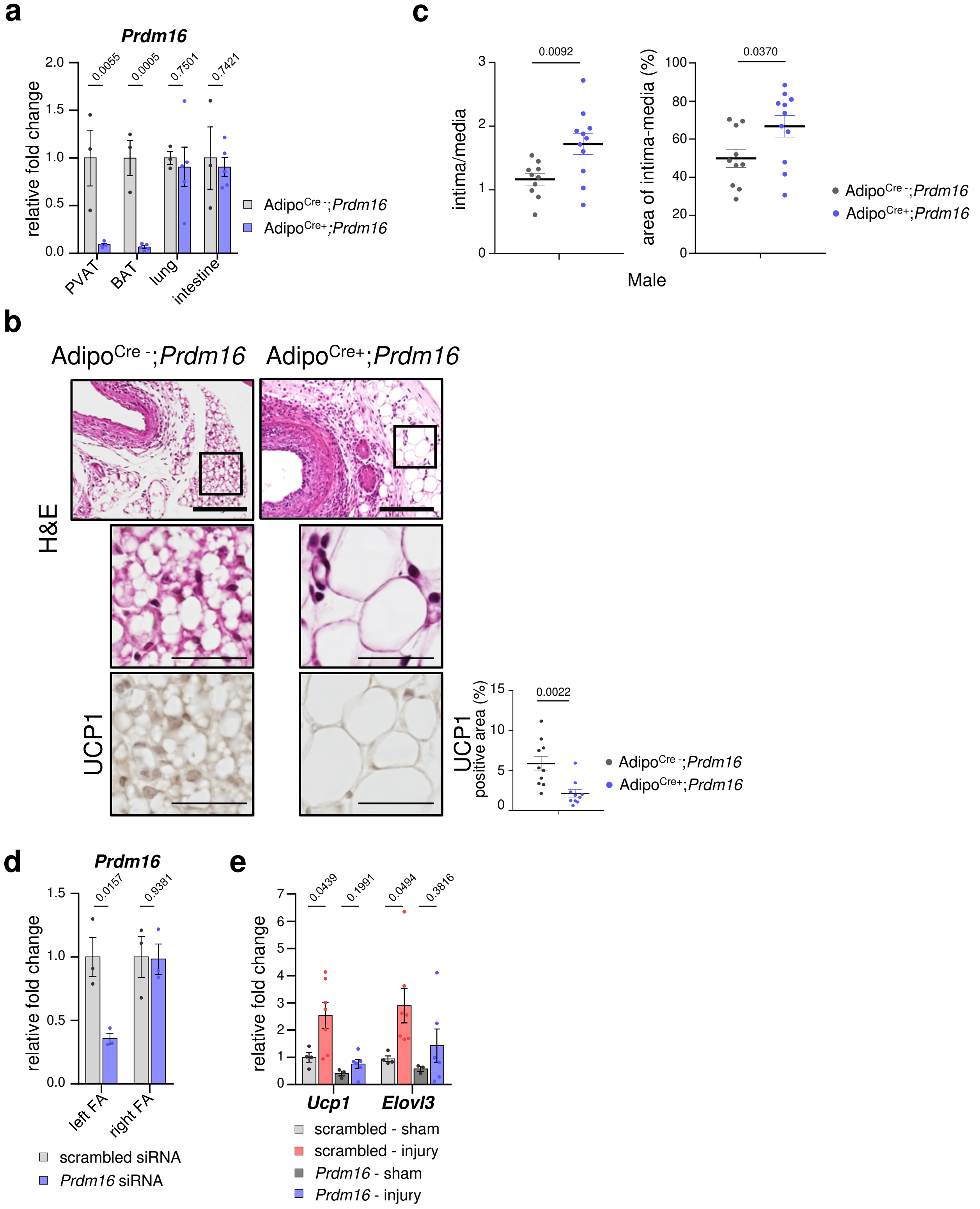

Supplementary Fig. 2 | Inhibition of PVAT beiging exacerbates vascular inflammation and remodeling.
(**a**) *Prdm16* expression in various tissues of Adipo^{Cre+};*Prdm16* mice compared with Adipo^{Cre-};*Prdm16* control littermates (Adipo^{Cre-};*Prdm16*, n = 3; Adipo^{Cre+};*Prdm16*, n = 5, two-tailed *t*-tests with Holm-Sidak's correction for multiple comparisons). (**b**) H&E and immunohistochemical staining for UCP1 in FAs 14 days after injury in Adipo^{Cre+};*Prdm16* mice compared with that in Adipo^{Cre-};*Prdm16* control littermates. Representative images are shown. Scale bars represent 100 µm (thick bars) and 50 µm (thin bars). The positive area of immunostaining in PVAT was analyzed (n = 10 for each group, unpaired two-tailed Student's *t*-test). (**c**) The ratio of intima to media area (intima/media) and the % intima–medial area in the region surrounded by the external elastic lamina (area of intima–media) 14 days after vascular injury in Adipo^{Cre+};*Prdm16* and Adipo^{Cre-};*Prdm16* (control) male mice were analyzed (Adipo^{Cre-};*Prdm16*, n = 10; Adipo^{Cre+};*Prdm16*, n = 11, unpaired two-tailed Student's *t*-test). (**d**) *Prdm16* expression in PVAT. siRNA of *Prdm16* or negative control (scrambled) was delivered to left FAs, and outer tissues surrounding FA were harvested from both sides of FA 48 h after delivery. Outer tissues surrounding right FAs were used as untreated controls (n=3 for each group, two-tailed *t*-tests with Holm-Sidak's correction for multiple comparisons). (**e**) Gene expression of BAT/beige adipocyte markers in PVAT 48 h after vascular injury. Vascular injury-induced PVAT beiging was significantly attenuated following *Prdm16* siRNA treatment (scrambled – sham, n = 4; scrambled – injury, n = 7; *Prdm16* – sham, n = 3; Prdm16 – injury, n = 6, one-way ANOVA followed by Tukey–Kramer post-hoc test). Data represent mean ± SEM. Source data are provided as a Source Data file.

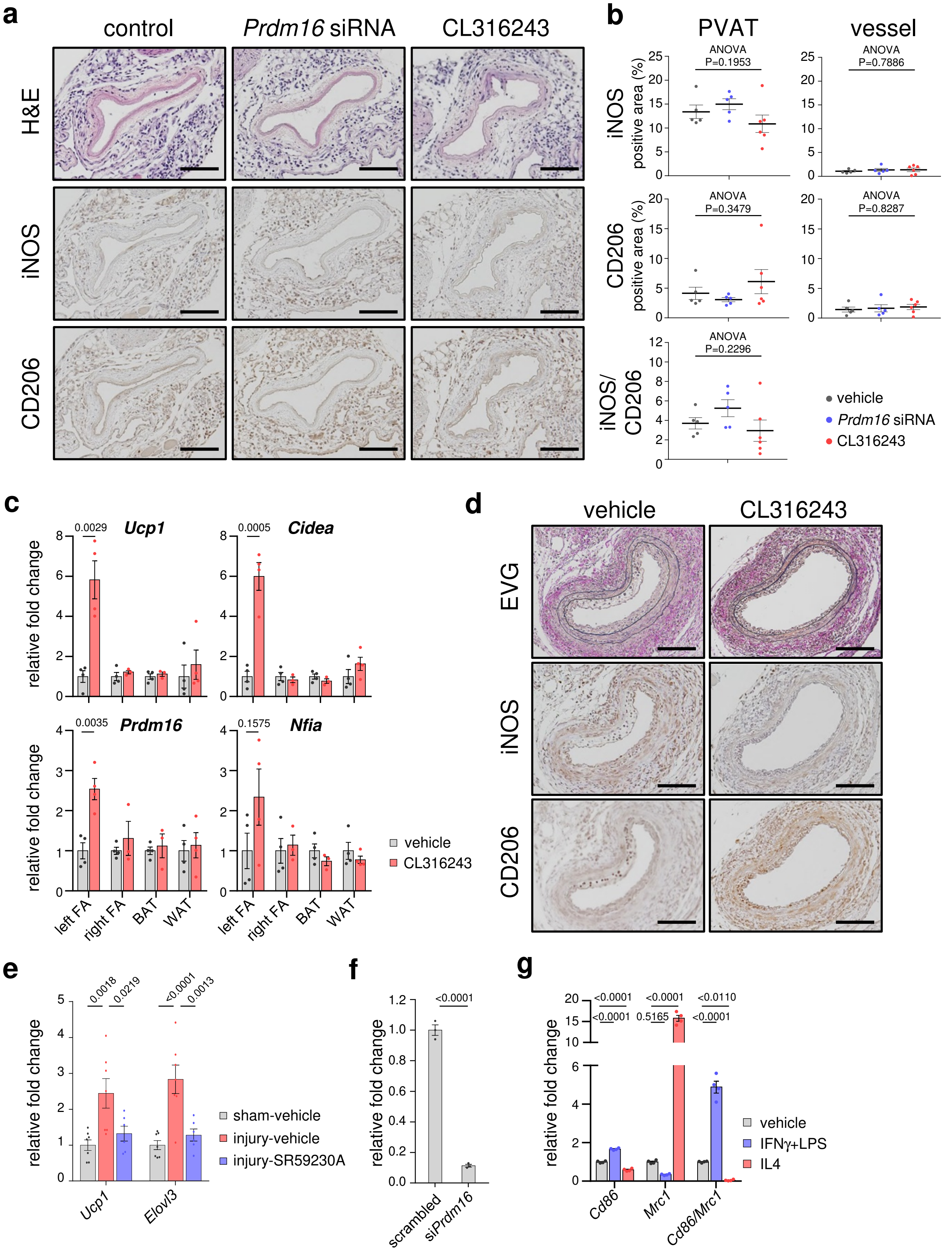

Supplementary Fig. 3 | Modification of PVAT beiging alters vascular inflammation and remodeling.
(**a**) H&E and immunohistochemical staining for iNOS and CD206 in FAs 3 days after injury in wild type mice treated with pluronic gel containing *Prdm16* siRNA or CL316243 applied to the PVAT surrounding the FAs. Scale bars represent 100 µm. Images are representative of three independent experiments. (**b**) The positive area of immunostaining in PVAT or arteries were analyzed (vehicle, n = 5; *Prdm16* siRNA, n = 5; CL316243, n =6, one-way ANOVA followed by Tukey–Kramer post-hoc test). (**c**) Gene expression of BAT/beige adipocyte markers in various adipose tissues. CL316243 or vehicle control was delivered to left FAs, and outer tissues surrounding FA were harvested from both sides of FA 48 h after delivery. Outer tissues surrounding right FAs were used as untreated controls, and intra-scapular BAT and parametrial WAT were also evaluated (left FA, n = 4; right FA, n = 3; BAT, n = 3; WAT, n =4, two-tailed *t*-tests with Holm-Sidak's correction for multiple comparisons). (**d**) EVG and immunohistochemical staining for iNOS and CD206 in FAs 14 days after injury in wild type mice treated with pluronic gel containing CL316243 or vehicle applied to the PVAT surrounding the FAs. Scale bars represent 100 µm. Images are representative of three independent experiments.

Supplementary Fig. 3 (continued) (**e**) Gene expression levels of BAT/beige markers (*Ucp1* and *Elovl3*) in PVAT at 24 h after injury in wild type mice treated with pluronic gel containing β3AR antagonist (SR59230A) or vehicle. (sham – vehicle, n = 7; injury – vehicle, n = 7; injury – SR59230A, n = 6, one-way ANOVA followed by Tukey–Kramer post-hoc test). (**f**) qRT-PCR analysis showing *Prdm16* gene expression in PVAT-preadipocytes introduced by scrambled or *Prdm16* siRNAs followed by stimulated with beige differentiation factors for 6 days (n = 3, biological replicates, representative data of three different culture lines are shown, unpaired two-tailed Student's *t*-test). (**g**) Phenotypic changes of macrophages in response to inflammatory stimuli. RAW 264.7 cells were treated with vehicle, IFNγ + LPS, or IL4 for 24 h. The results of qRT-PCR for the gene expression levels of macrophage phenotype markers are shown (n = 4 biological replicates, representative data are shown, one-way ANOVA followed by Tukey–Kramer post-hoc test). Data represent mean ± SEM. Source data are provided as a Source Data file.

a

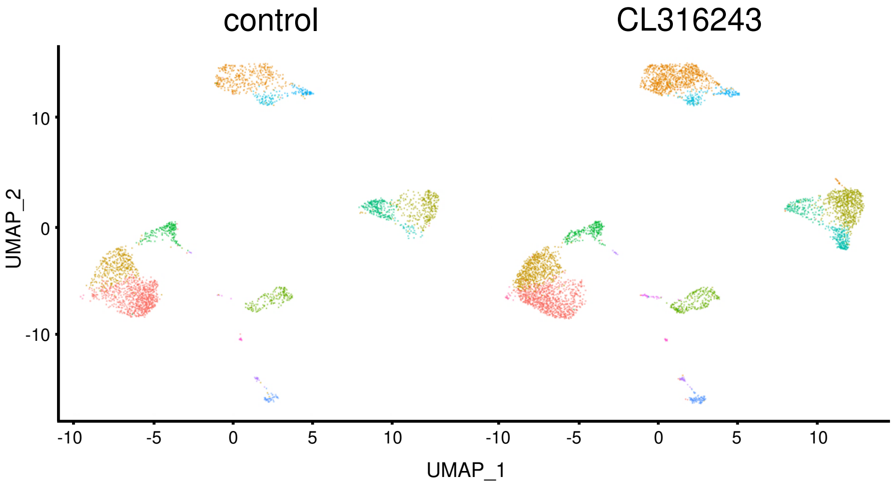

b

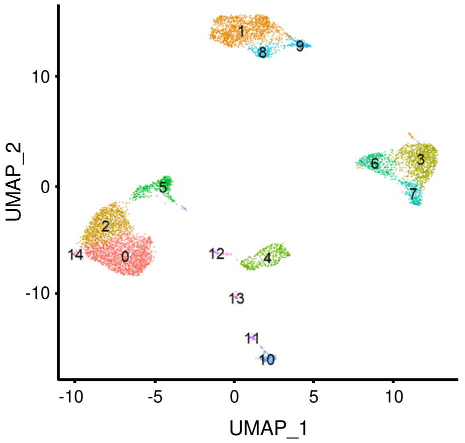

	Cell type	Representative genes
0	Endothelial cell 1	*Pecam1*
1	Fibroblast 1	*Col3a1, Htra3*
2	Endothelial cell 2	*Pecam1*
3	White adipocyte 1	*Plin4, Retn, Cfd*
4	Dendritic cell/Macrophage	*Cd83, Mrc1, Cd163*
5	Smooth muscle cell	*Myh11*
6	White adipocyte 2	*Plin4, Retn, Cfd*
7	Beige adipocyte	*Ucp1, Cidea, Ppargc1a*
8	Fibroblast 2	*Col3a1, Htra3*
9	Fibroblast 3	*Col3a1, Htra3*
10	Nerve cell 1	*Nrxn3*
11	Mast cell	*Kit*
12	Endothelial cell 3	*Pecam1*
13	Nerve cell 2	*Mbp, Plp1*
14	Endothelial cell 4	*Pecam1*

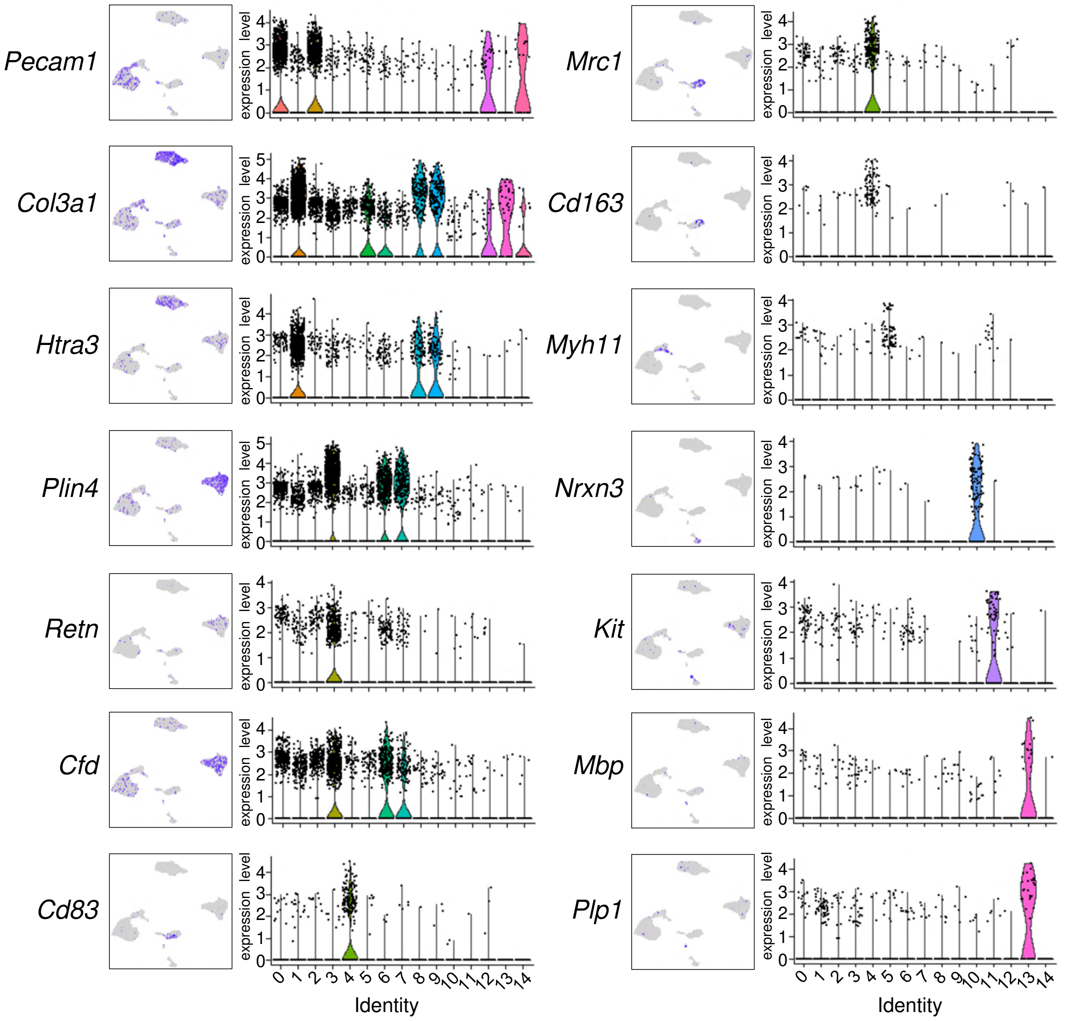

Supplementary Fig. 4

Supplementary Fig. 4 | Characterization of inguinal WAT cell subtypes treated with CL316243.

(**a**) Unbiased clustering analysis by UMAP dimensionality reduction using scRNA-seq data of mouse inguinal WAT treated with CL316243 or control. The integration step was performed with Seurat. Integrated UMAP plot was split by different condition. C7 was predominantly composed of CL316243-treated cells. (**b**) UMAP visualization and marker-based annotation of cell subtypes. Feature and violin plots of representative genes of each cluster are shown. The plots of *Ucp1*, *Cidea* and *Ppargc1a* are shown in Fig. 4c.

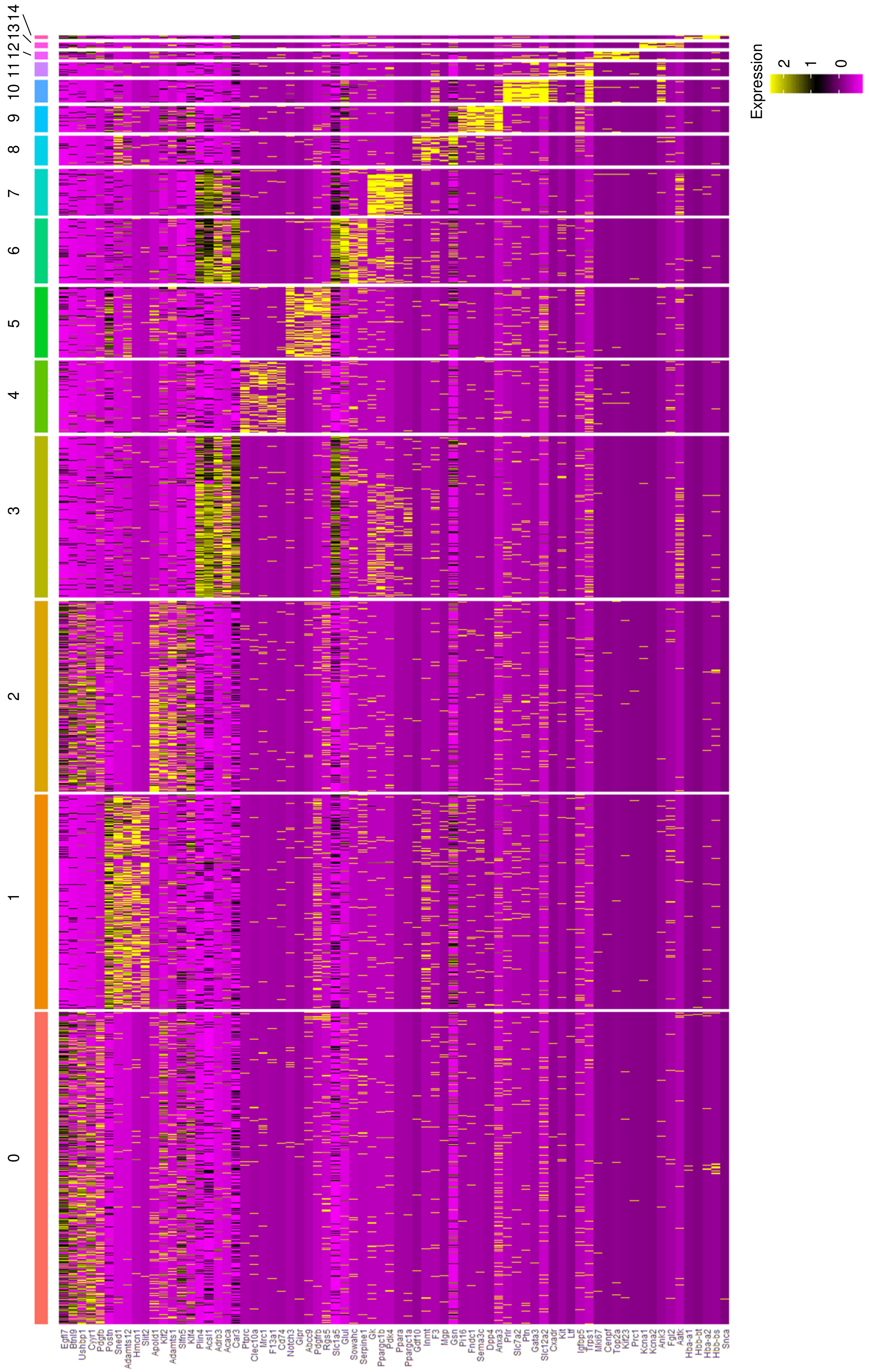

Supplementary Fig. 5 | scRNA-seq of WAT treated with CL316243.

Heat map of signature genes from scRNA-seq of mouse inguinal WAT treated with CL316243 or control, characterizing expression of cell type-defining genes in each cluster. Cells were divided into 15 clusters.

a

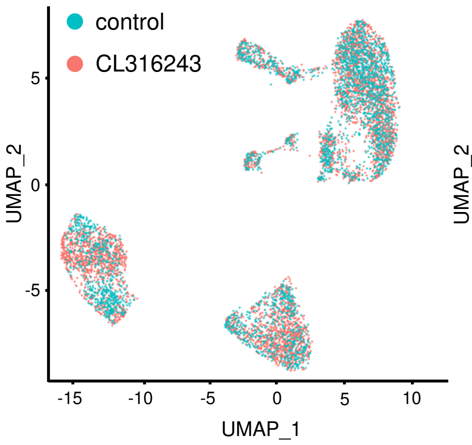

b

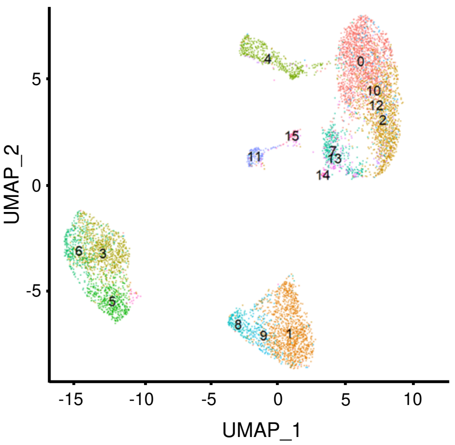

	Cell type	Representative genes
0	Endothelial cell 1	*Pecam1*
1	Fibroblast 1	*Col3a1, Htra3*
2	Endothelial cell 2	*Pecam1*
3	White adipocyte 1	*Plin4, Retn, Cfd*
4	Dendritic cell/Macrophage	*Cd83, Mrc1, Cd163*
5	White adipocyte 2	*Plin4, Retn, Cfd*
6	Beige adipocyte	*Ucp1, Cidea, Ppargc1a*
7	Endothelial cell 4	*Pecam1*
8	Fibroblast 2	*Col3a1, Htra3*
9	Fibroblast 3	*Col3a1, Htra3*
10	Endothelial cell 3	*Pecam1*
11	Nerve cell 1	*Nrxn3*
12	Endothelial cell 5	*Pecam1*
13	Smooth muscle cell	*Myh11*
14	Nerve cell 2	*Mbp, Plp1*
15	Mast cell	*Kit*

c

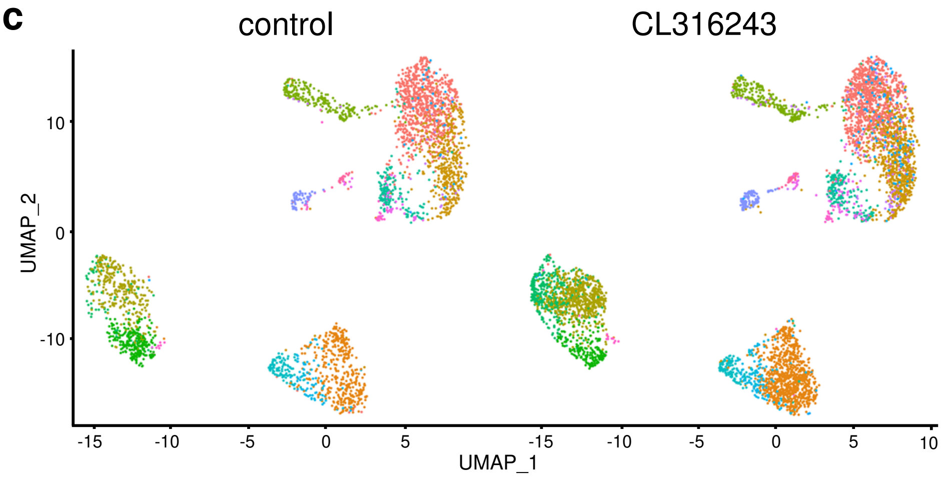

d

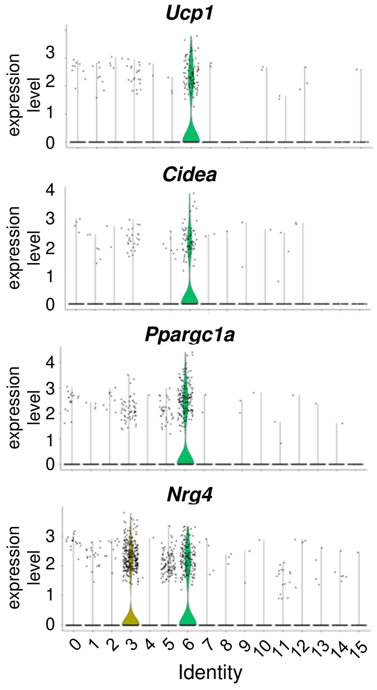

e

Term	*P* value	Representative genes
lipid metabolic process	9.12E-16	*Acsl1 Plin1 Ppara*
gluconeogenesis	3.04E-04	*Gpd1 Pck1 Ppargc1a*
tricarboxylic acid cycle	4.23E-04	*Cs Aco2 Idh3a*
fatty acid beta-oxidation using acyl-CoA dehydrogenase	0.00414	*Acox1 Etfdh Acadm*
receptor binding	0.00575	*Nrg4 Lpl Scp2*
brown fat cell differentiation	0.0129	*Adrb3 Arl4a Sh2b2*
carbohydrate metabolic process	0.0210	*Gk Pdk4*

f

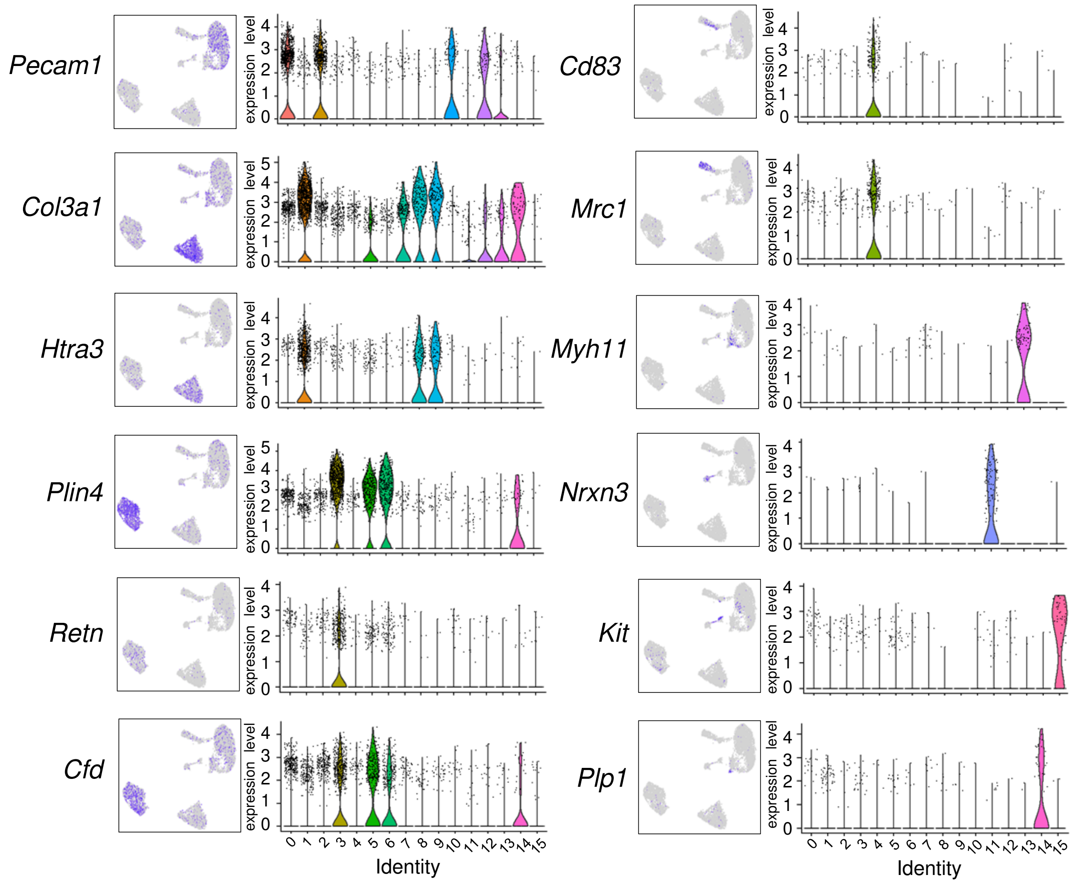

Supplementary Fig. 6

Supplementary Fig. 6 | Inguinal WAT cells treated with CL316243 show characteristic clusters independent of the means of integration (integrated with Harmony).

(**a**–**c**) Clustering analysis of UMAP dimensionality reduction using scRNA-seq data from publicly available datasets of mouse iWAT treated with CL316243 or control (GSE 133486). The integration step was performed with Harmony. Adipose tissue cells were divided into 16 clusters that were color-coded by cell type (**b**) and by stimulation (CL316243 treatment [pink] or control [turquoise]) (**a**). The integrated UMAP plot was split into different conditions. C6 was predominantly composed of CL316243-treated cells (**c**). (**d**) Violin plots of representative beige/BAT marker genes and *Nrg4* in each cluster indicated in (**b**). (**e**) Gene ontology analysis of the top 100 regulated genes in C6 showing that several biological processes annotated by the genes were significantly changed; *Nrg4* is involved in receptor-binding. *P*-values are determined by Fisher's Exact test. (**f**) Marker-based annotation of cell subtypes. Feature and violin plots of the representative genes of each cluster are shown.

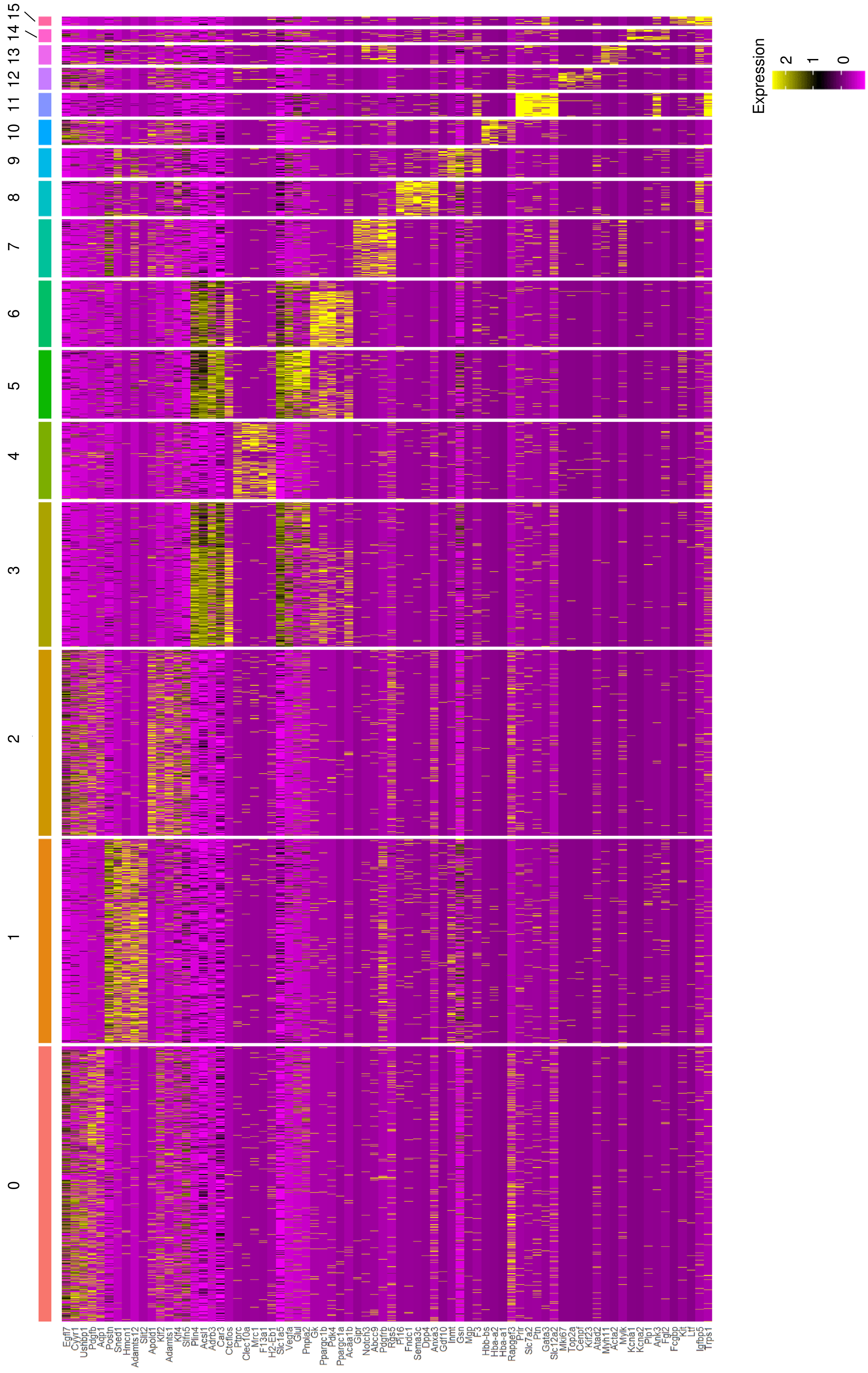

Supplementary Fig. 7 | scRNA-seq of WAT treated with CL316243 (integrated with Harmony).
Heat map of signature genes from scRNA-seq of mouse inguinal WAT treated with CL316243 or control, characterizing expression of cell type-defining genes in each cluster. Integration was performed with Harmony. The cells were divided into 16 clusters.

a

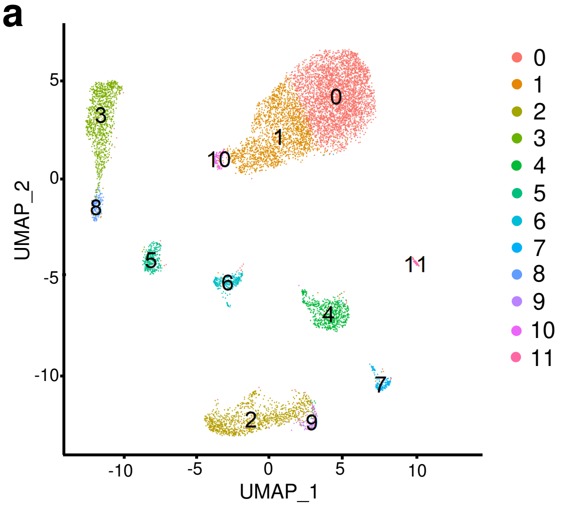

	Cell type	Representative genes
0	Endothelial cell 1	*Pecam1*
1	Endothelial cell 2	*Pecam1*
2	White adipocyte	*Plin4, Retn, Cfd*
3	Fibroblast 1	*Col3a1, Htra3*
4	Smooth muscle cell	*Myh11*
5	Dendritic cell/Macrophage	*Cd83, Mrc1, Cd163*
6	Nerve cell 1	*Nrxn3*
7	Mast cell	*Kit*
8	Fibroblast 2	*Col3a1, Htra3*
9	Beige adipocyte	*Ucp1, Cidea, Ppargc1a*
10	Endothelial cell 3	*Pecam1*
11	Nerve cell 2	*Mbp, Plp1*

b

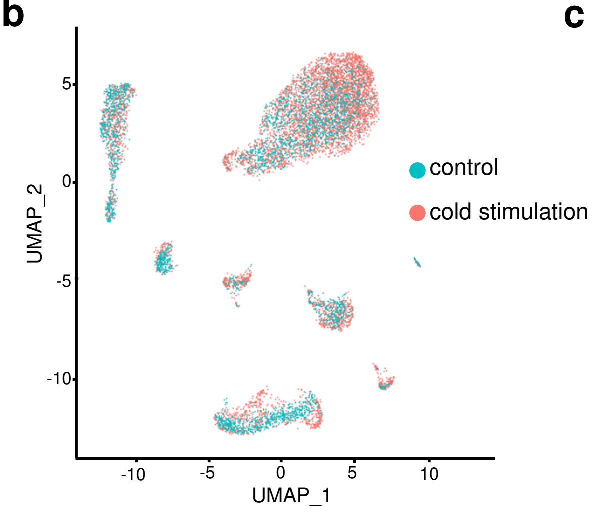

c

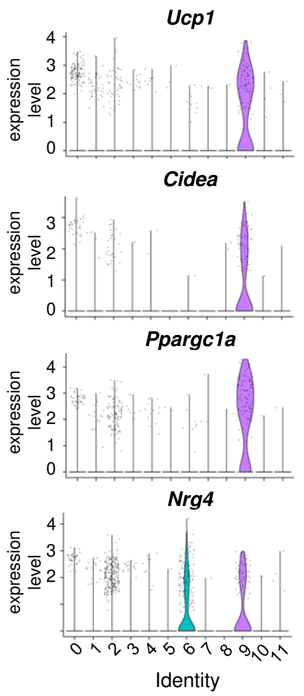

d

Term	*P* value	Representative genes
lipid metabolic process	9.12E-16	*Acsl1 Plin1 Cidea*
tricarboxylic acid cycle	3.29E-07	*Cs Aco2 Idh3a*
fatty acid beta-oxidation using acyl-CoA dehydrogenase	0.00414	*Acox1 Etfdh Acadvl*
kinase activity	0.00792	*Gk Pdk4*
receptor binding	0.0198	*Nrg4 Lpl Acox1*
response to temperature stimulus	0.0301	*Ucp1 Acot11*

e

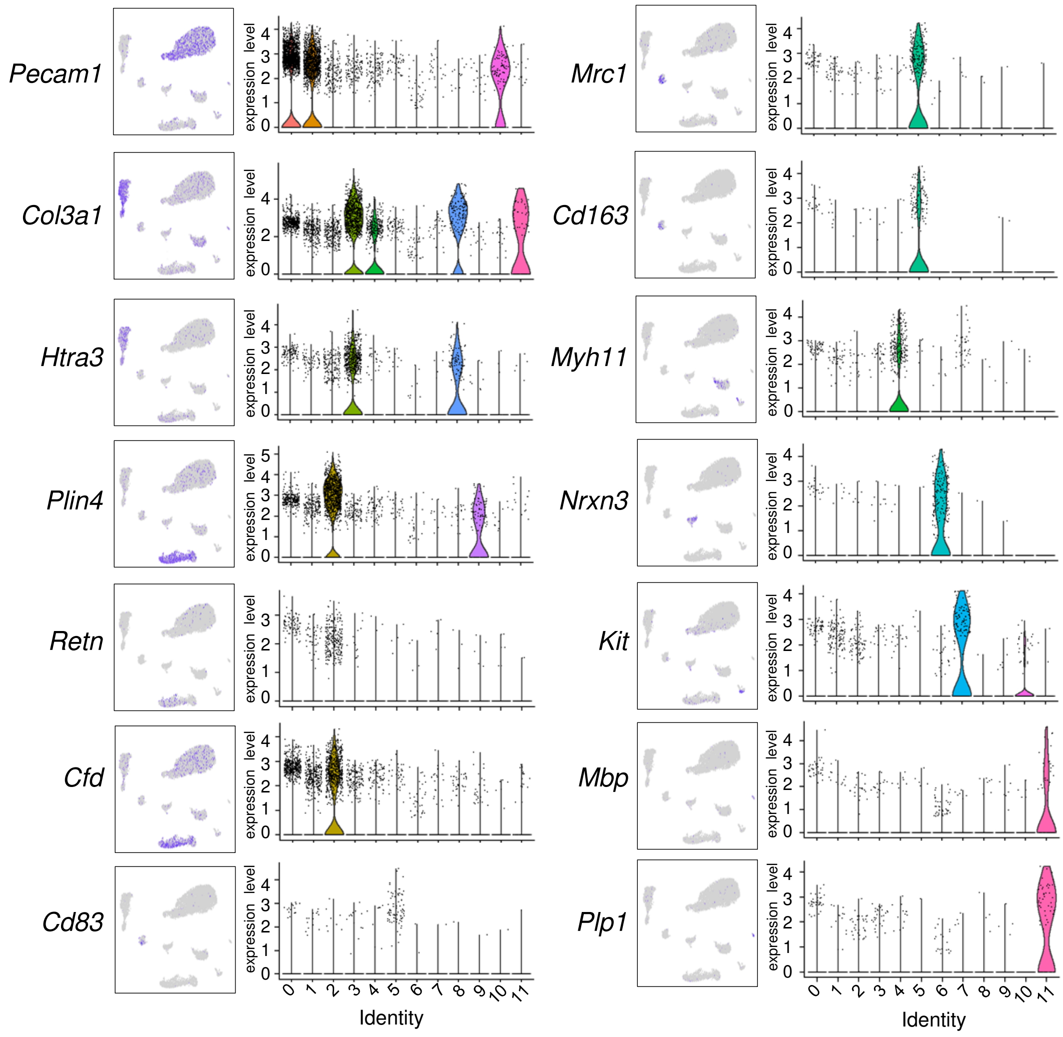

Supplementary Fig. 8

Supplementary Fig. 8 | Characterization of inguinal WAT cell subtypes treated with cold stimulation.
(**a, b**) Clustering analysis of UMAP dimensionality reduction using scRNA-seq data in publicly available datasets of mouse iWAT treated with cold stimulation or control (GSE 133486), which divided adipose tissue cells into 12 clusters that are color-coded by cell type (**a**) and by stimulation method (cold stimulation [pink] or control [turquoise]) (**b**). C9 was predominantly composed of cold-stimulated cells. (**c**) Violin plots of representative beige/BAT marker genes and *Nrg4* in each cluster indicated in (**a**). (**d**) Gene ontology analysis for the top 100 regulated genes in C9 showing that several biological processes annotated by the genes were changed significantly. *Nrg4* was listed as a gene involved in receptor-binding. *P*-values are determined by Fisher's Exact test. (**e**) Feature and violin plots of representative genes of each cluster are shown.

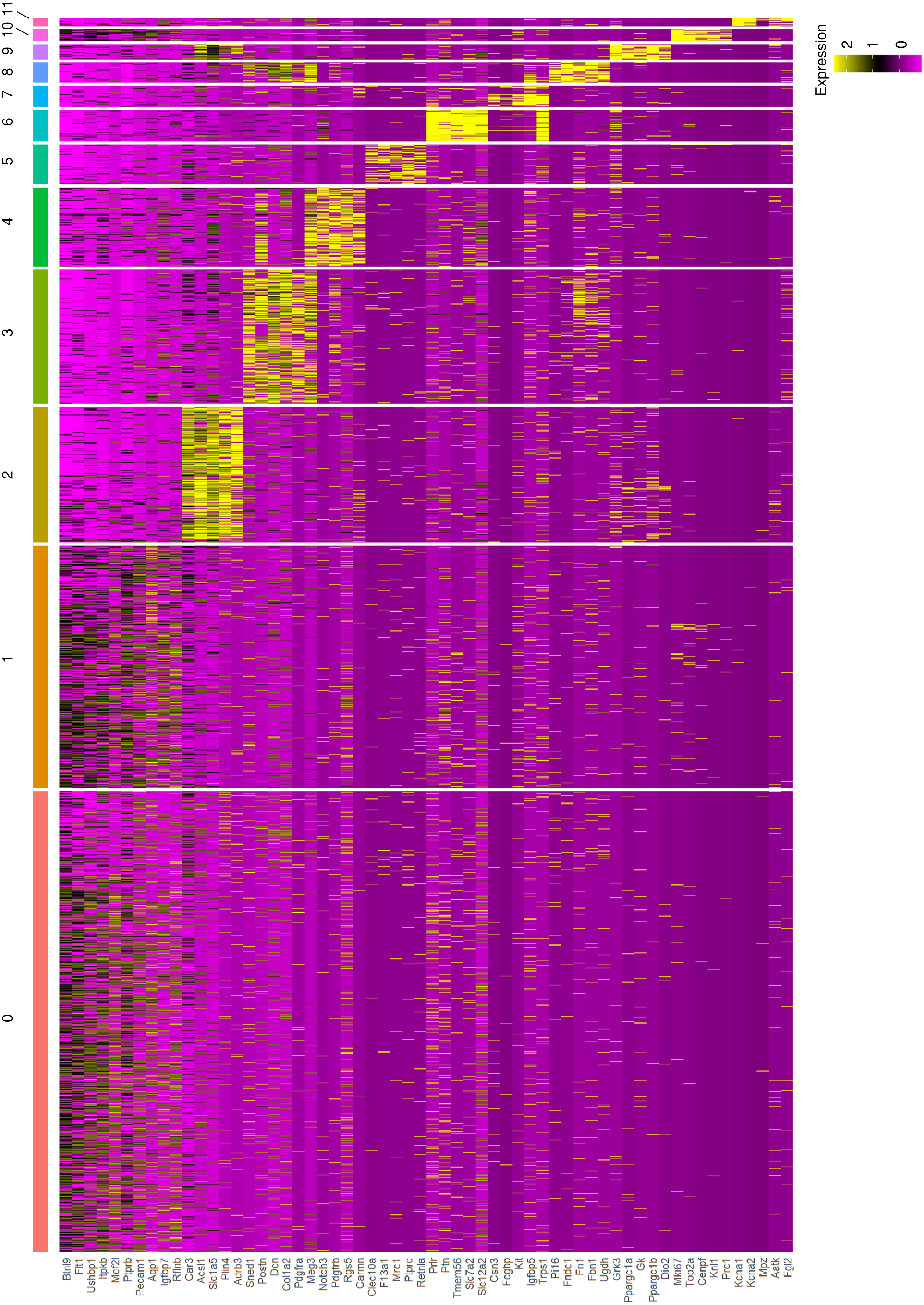

Supplementary Fig. 9 | scRNA-seq of WAT treated with cold stimulation.
Heat map of signature genes from scRNA-seq of mouse inguinal WAT treated with cold stimulation or control, characterizing the expression of cell type-defining genes in each cluster. The cells were then divided into 12 clusters.

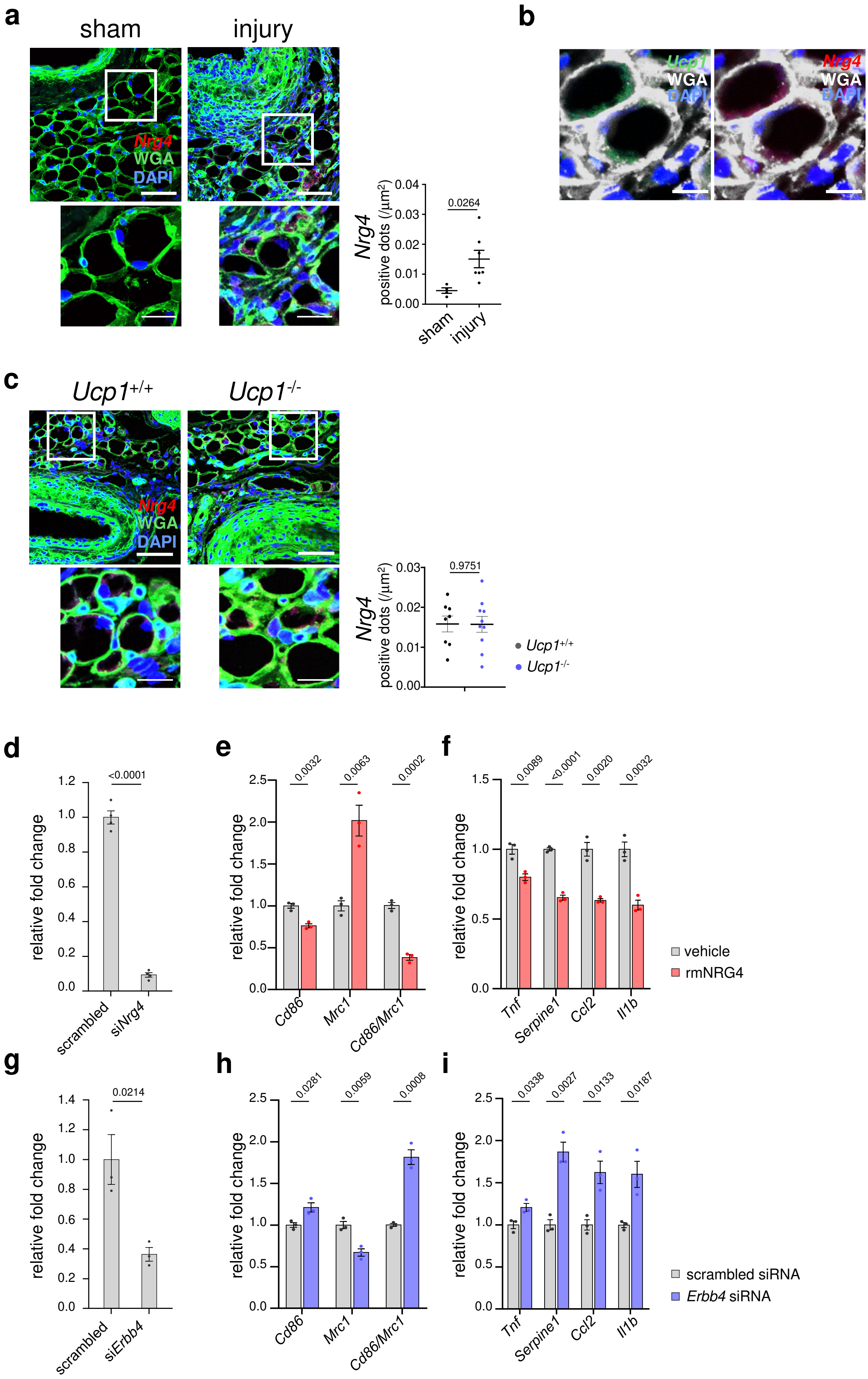

Supplementary Fig. 10 | Effect of Nrg4 on the polarization of macrophages and identification of Nrg4 expression in vivo. (**a**) *In situ* hybridization showing *Nrg4* mRNA expression in PVAT surrounding the injured FAs of wild type mice 14 days after injury compared with sham-operated FAs. Representative images are shown (sham, n = 4; injury, n =7, unpaired two-tailed Student's *t*-test). Scale bars represent 50 µm (thick bars) and 20 µm (thin bars). (**b**) *In situ* hybridization showing *Ucp1* and *Nrg4* mRNA expression 14 days after vascular injury in wild type mice. Representative images are shown (n = 3 for each group). Scale bars represent 10 µm. (**c**) *In situ* hybridization showing *Nrg4* mRNA expression 14 days after vascular injury in *Ucp1*$^{+/+}$ and *Ucp1*$^{-/-}$ mice. Representative images are shown (*Ucp1*$^{+/+}$, n = 8; *Ucp1*$^{-/-}$, n = 10, unpaired two-tailed Student's *t*-test). Scale bars represent 50 µm (thick bars) and 20 µm (thin bars).

Supplementary Fig. 10 (continued) (**d**) qRT-PCR analysis showing *Nrg4* gene expression in PVAT-preadipocytes introduced by scrambled or *Nrg4* siRNAs followed by stimulated with beige differentiation factors for 6 days (n = 4, biological replicates, representative data of three different culture lines are shown, unpaired two-tailed Student's *t*-test). (**e, f**) RAW 264.7 cells were pre-treated with IFNγ and LPS for 24 h and treated with recombinant murine (rm) NRG4 (100 ng/ml), and mRNA was extracted 48 h after treatment. Results of qRT-PCR analyses of the gene expression levels of macrophage phenotype markers (**e**) and inflammatory cytokines (**f**) are shown (n = 3, biological replicates, representative data of three different culture lines are shown, two-tailed *t*-tests with Holm-Sidak's correction for multiple comparisons). (**g**) qRT-PCR analysis showing *Erbb4* gene expression in PVAT-preadipocytes introduced by scrambled or *Erbb4* siRNAs followed by stimulated with beige differentiation factors for 6 days (n = 3, biological replicates, representative data of three different culture lines are shown, unpaired two-tailed Student's *t*-test). (**h, i**) PVAT-preadipocytes were introduced by siRNA of *Erbb4* or scrambled and stimulated with beige differentiation factors. Culture media conditioned by these cells were added to RAW 264.7 cells and mRNA was extracted 48 h after treatment. Results of qRT-PCR analyses of the gene expression levels of macrophage phenotype markers (**h**) and inflammatory cytokines (**i**) are shown (n = 3, biological replicates, representative data of three different culture lines are shown, two-tailed *t*-tests with Holm-Sidak's correction for multiple comparisons). Data represent mean ± SEM. Source data are provided as a Source Data file.

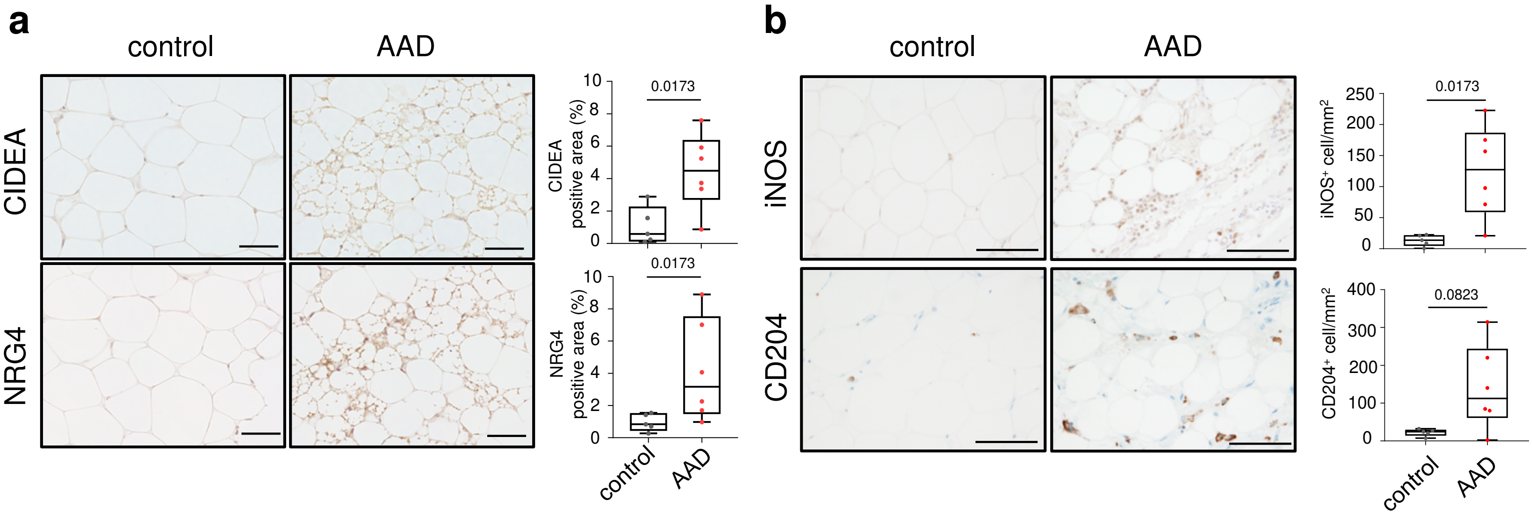

Supplementary Fig. 11 | Immunohistochemical staining of PVAT in human aorta with acute dissection.

(a) Immunohistochemical staining for CIDEA (top) and NRG4 (bottom) was performed in the aortic PVAT of patients with or without AAD. Representative images are shown. Scale bars represent 100 µm. The % of CIDEA$^+$ and NRG4$^+$ areas in the PVAT from patients with or without AAD were analyzed (control, n = 5; AAD, n = 6, two-sided Mann–Whitney U-test. **(b)** Immunohistochemical staining for iNOS (top; M1 marker) and CD204 (bottom; M2 marker) was performed in the aortic PVAT of patients with or without AAD. Representative images are shown. Scale bars represent 100 µm. The numbers of iNOS$^+$ and CD204$^+$ cells in the PVAT from patients with or without AAD were analyzed (control, n = 5; AAD, n = 6, two-sided Mann–Whitney U-test). Box plots denote the median, 25–75th percentiles, and minimum and maximum values. Source data are provided as a Source Data file.

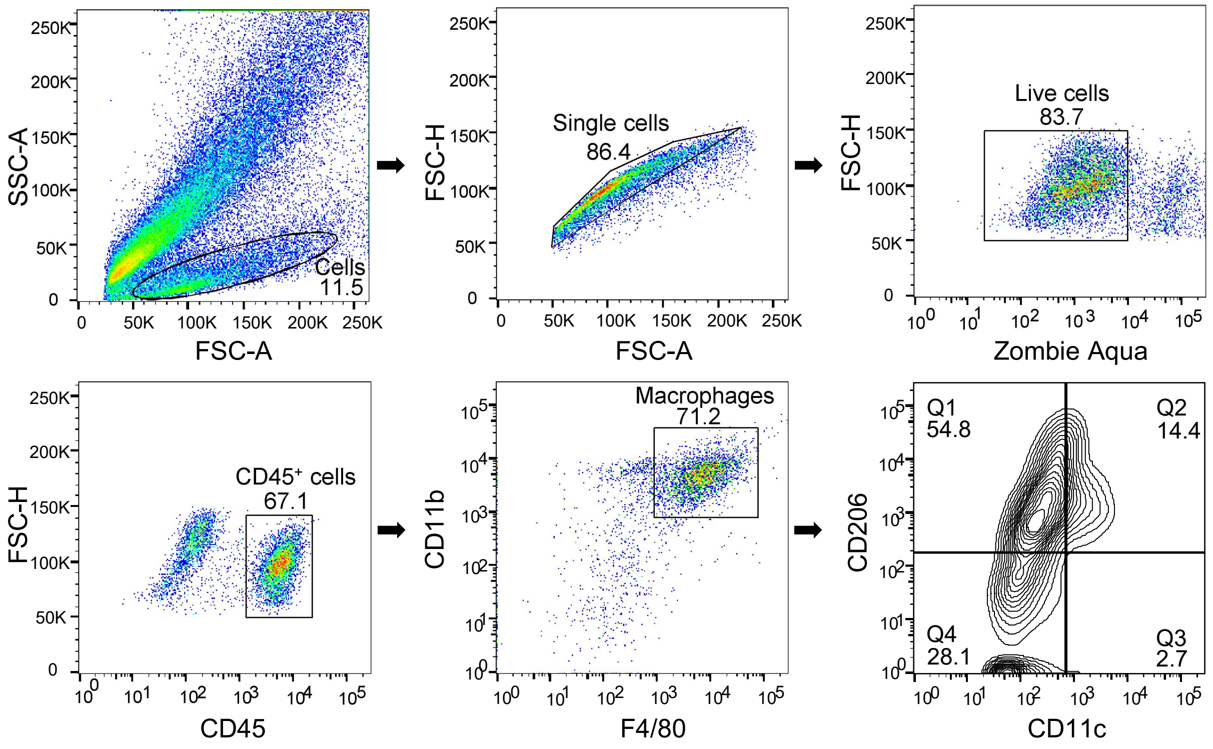

Supplementary Fig. 12 | PVAT macrophage switching in AAD murine model treated with CL316243.

A representative gating strategy to analyze PVAT infiltration of macrophages ($CD45^+$, $CD11b^+$, and $F4/80^+$ mononuclear cells) was presented, which could further differentiate into classically activated (M1; defined as $CD11c^+$ and $CD206^-$) and alternatively activated (M2; defined as $CD11c^-$ and $CD206^+$) macrophages.

Supplementary Table 1: List of sequences of qRT-PCR primers.

Genes	Primer forward	Primer reverse
Adgre1	CTTTGGCTATGGGCTTCCAGTC	GCAAGGAGGACAGAGTTTATCGTG
B2m	CATGGCTCGCTCGGTGAC	CAGTTCAGTATGTTCGGCTTCC
Ccl2	CCACTCACCTGCTGCTACTCAT	TGGTGATCCTCTTGTAGCTCTCC
Cd11b	ATGGACGCTGATGGCAATACC	TCCCCATTCACGTCTCCCA
Cd11c	CTGGATAGCCTTTCTTCTGCTG	GCACACTGTGTCCGAACTC
Cd4	TCCTTCCCACTCAACTTTGC	AAGCGAGACCTGGGGTATCT
Cd68	ACTTCGGGCCATGTTTCTCT	GCTGGTAGGTTGATTGTCGT
Cd8	GCTCAGTCATCAGCAACTCG	ATCACAGGCGAAGTCCAATC
Cd80	GGCAAGGCAGCAATACCTTA	CTCTTTGTGCTGCTGATTCG
Cd86	TCTCCACGGAAACAGCATCT	CTTACGGAAGCACCCATGAT
Cfd	CATGCTCGGCCCTACATGG	CACAGAGTCGTCATCCGTCAC
Cidea	GGCCGTGTTAAGGAATCTGC	CATGAACCAGCCTTTGGTGC
Cox8b	CCAGCCAAAACTCCCACTT	GCTCTCCAAGTGGGCTAAGA
Elovl3	CCAACAACGATGAGCAACAG	CGGGTTAAAAATGGACCTGA
Fcer1a	TGTGTACTTGAATGTAACGCAAGA	GGACTAAGACCATGTCAGCAGAT
Gapdh	CACTGAAGGGCATCTTGG	CATTGTCATACCAGGAAATGAG
Il10	CTTACTGACTGGCATGAGGATCA	GCAGCTCTAGGAGCATGTGG
Il1a	TTGGTTAAATGACCTGCAACA	GAGCGCTCACGAACAGTTG
Il1b	AGTTGACGGACCCCAAAAG	AGCTGGATGCTCTCATCAGG
Il6	CTGCAAGAGACTTCCATCCAG	AGTGGTATAGACAGGTCTGTTGG
Mrc1	CAGGTGTGGGCTCAGGTAGT	TGTGGTGAGCTGAAAGGTGA
Nfia	CCATTTTACACAGGCCAAGG	TGGCTGGGTGTGAGAAGTAAG
Nrg4	TCTGTCGGCAGCTTTCGT	CTGAAGGTGGCCCTTCCT
Ppargc1a	ATAGAGTGTGCTGCTCTGGTTGGT	TGGTCGCTACACCACTTCAATCCA
Prdm16	CAGCACGGTGAAGCCATTC	GCGTGCATCCGCTTGTG
Rstn	AAGAACCTTTCATTTCCCCTCCT	GTCCAGCAATTTAAGCCAATGTT
Serpine1	CCTCCTCATCCTGCCTAAGTT	GGCCAGGGTTGCACTAAAC
Tnf	TCTTCTCATTCCTGCTTGTGG	GAGGCCATTTGGGAACTTCT
Ucp1	GGCCTCTACGACTCAGTCCA	TAAGCCGGCTGAGATCTTGT

Supplementary Table 2: Deconvolution of bulk RNA-seq dataset with scRNA-seq data as reference.

	iWAT_Young_Ctrl_1	iWAT_Young_Ctrl_2	iWAT_Young_Ctrl_3	iWAT_Young_Ctrl_4	iWAT_Young_CL_1	iWAT_Young_CL_2	iWAT_Young_CL_3	iWAT_Young_CL_4
Endothelial cell 1	0.4703307117	0.5049030726	0.4734915060	0.4651025981	0.4296044583	0.4980807401	0.4023037369	0.4991476032
Fibroblast 1	0.0000000000	0.0000000000	0.0000000000	0.0000000000	0.0000000000	0.0000000000	0.0000000000	0.0000000000
Endothelial cell 2	0.0000000000	0.0000000000	0.0000000000	0.0000000000	0.0000000000	0.0000000000	0.0000000000	0.0000000000
White adipocyte 1	0.2550167726	0.1482379674	0.1414281870	0.1239650753	0.2827130428	0.2473239968	0.2975312132	0.2874352377
Dendritic cell/Macrophage	0.1455949827	0.1868209184	0.2370663733	0.2772765226	0.1564755034	0.1070411641	0.1562212407	0.0662757281
Smmoth muscle cell	0.0000000000	0.0000000000	0.0000000000	0.0000000000	0.0000000000	0.0000000000	0.0000000000	0.0000000000
White adipocyte 2	0.0000000000	0.0000000000	0.0000000000	0.0000000000	0.0001593604	0.0000000000	0.0000000000	0.0000000000
Beige adipocyte	0.0000000000	0.0000000000	0.0000000000	0.0000000000	0.0000000000	0.0025891186	0.0021396151	0.0398656897
Fibroblast 2	0.0203786891	0.0370084143	0.0176314840	0.0105657876	0.0092600330	0.0147060124	0.0121655181	0.0000000000
Fibroblast 3	0.0000000000	0.0000000000	0.0000000000	0.0000000000	0.0000000000	0.0000000000	0.0000000000	0.0000000000
Nerve cell 1	0.0001454120	0.0001492390	0.0001254499	0.0000000000	0.0000000000	0.0000562746	0.0002685761	0.0000840551
Mast cell	0.0719125826	0.0817470869	0.0903500269	0.0903618382	0.0760630641	0.0797972573	0.0884700030	0.0705118886
Endothelial cell 3	0.0000000000	0.0000000000	0.0000000000	0.0010054823	0.0008274477	0.0000000000	0.0000000000	0.0000000000
Nerve cell 2	0.0090790518	0.0132871809	0.0131848125	0.0133927474	0.0065742105	0.0096901079	0.0075279701	0.0037327993
Endothelial cell 4	0.0275417975	0.0278461206	0.0267221604	0.0183299486	0.0383228798	0.0407153281	0.0333721268	0.0329469984

Predicted proportions of cell fractions across bulk RNA-seq libraries from murine inguinal WAT (iWAT) treated with CL316243 or control, performed with the R package MuSiC. Source data are provided as a Source Data file.
